# OM-85 is an immunomodulator of interferon-β production and inflammasome activity

**DOI:** 10.1038/srep43844

**Published:** 2017-03-06

**Authors:** A. T. Dang, C. Pasquali, K. Ludigs, G. Guarda

**Affiliations:** 1 Department of Biochemistry, University of Lausanne, Epalinges, Switzerland; 2 Vifor-Pharma c/o OM Pharma SA, 1217 Meyrin 1/Geneva, Switzerland; 3 Present address: Roche Pharma (Schweiz) AG, Schönmattstrasse 2, 4153 Reinach, Switzerland

## Abstract

The inflammasome–IL-1 axis and type I interferons (IFNs) have been shown to exert protective effects upon respiratory tract infections. Conversely, IL-1 has also been implicated in inflammatory airway pathologies such as asthma and chronic obstructive pulmonary disease (COPD). OM-85 is a bacterial extract with proved efficacy against COPD and recurrent respiratory tract infections, a cause of co-morbidity in asthmatic patients. We therefore asked whether OM-85 affects the above-mentioned innate immune pathways. Here we show that OM-85 induced interferon-β through the Toll-like receptor adaptors Trif and MyD88 in bone marrow-derived dendritic cells. Moreover, it exerted a dual role on IL-1 production; on the one hand, it upregulated proIL-1β and proIL-1α levels in a MyD88-dependent manner without activating the inflammasome. On the other hand, it repressed IL-1β secretion induced by alum, a well-known NLRP3 activator. *In vivo*, OM-85 diminished the recruitment of inflammatory cells in response to peritoneal alum challenge. Our findings therefore suggest that OM-85 favors a protective primed state, while dampening inflammasome activation in specific conditions. Taken together, these data bring new insights into the mechanisms of OM-85 action on innate immune pathways and suggest potential explanations for its efficacy in the treatment of virus-induced airway diseases.

Inflammatory airway diseases are an emerging health problem with high incidence in developed countries. Two important examples are chronic obstructive pulmonary disease (COPD) and asthma. Both disorders are characterized by narrowing of the airways and by a strong inflammatory component^1^. Multiple mediators have been implicated in the development of these pathologies, as in particular T helper type (Th)2 cytokines^1^. However, significant alterations of innate immune cytokines have also been associated with these disorders. Asthmatic patients show decreased production of type I and III interferons (IFNs), which augments susceptibility to virally induced asthma exacerbations^2^,^3^,^4^,^5^,^6^. IFNs are important antiviral and immunomodulatory cytokines, promoting control of respiratory infections while reducing collateral damage and excessive inflammation^2^,^3^,^4^,^5^,^6^,^7^,^8^,^9^,^10^,^11^,^12^. In fact, viral infections early in life, but also at later stages, are increasingly recognized as predisposing or aggravating events of asthmatic manifestations^13^,^14^,^15^.

Interestingly, also several links between interleukin (IL)-1 and inflammatory airway diseases have been established^16^,^17^. IL-1α and IL-1β are strong inflammatory cytokines, sharing the common IL-1 receptor. Whereas IL-1α does not require processing for activity, IL-1β is synthesized as a biologically inactive precursor, called proIL-1β, and requires cleavage by the protease caspase-1 to be converted into its active form^18^. The activation of caspase-1 takes place within a protein complex known as “inflammasome”, which is formed by a nucleating sensor protein, an adaptor protein called apoptosis-associated speck-like protein containing a CARD (ASC), and caspase-1^18^,^19^. Several receptors of the NOD-like receptor (NLR) family have been shown to assemble inflammasome platforms, including the intensively studied NLRP3^19^,^20^. NLRP3 is well expressed and best studied in innate immune cells and senses a plethora of stimuli encompassing particulate stimuli, pore-forming toxins, and pathogens of fungal, bacterial, as well as viral origin^18^,^19^,^20^,^21^. In addition, AIM2, which is mainly expressed in immune cells, nucleates a well-characterized inflammasome upon recognition of cytoplasmic double-stranded DNA^18^,^22^.

These two sensors, are of particular interest with regard to antiviral and inflammatory reactions in the lung^16^,^23^,^24^,^25^,^26^,^27^. Upon influenza infection, NLRP3 inflammasome activation in lung dendritic cells (DCs) and macrophages exerts a protective effect, whereas its overactivation contributes to severe pathophysiology^23^,^24^,^25^,^26^,^27^. Similarly, in models of pulmonary fibrosis and silicosis, a detrimental role for the NLRP3/IL-1 axis was witnessed^16^,^28^,^29^,^30^,^31^,^32^,^33^,^34^,^35^,^36^,^37^,^38^,^39^. The overall contribution of inflammasome and IL-1 to airway pathologies remains therefore multifaceted, exerting protective effects in the context of viral infections, but also harmful ones in chronic inflammatory conditions.

From the medical standpoint, recurrent respiratory tract infections (RTIs), COPD, as well as wheezing and asthma are major public health problems. Concomitantly, prevalence of antibiotic resistance among common respiratory pathogens has recently risen, highlighting the need to develop strategies of immunization against respiratory bacteria and viruses^40^,^41^,^42^. Launched more than 30 years ago, OM-85 (Broncho-Vaxom^®^, Broncho-Munal^®^, Ommunal^®^, Paxoral^®^, Vaxoral^®^), an oral medicine of biological origin used for the prevention of recurrent RTIs and/or exacerbations in at-risk populations, was shown to be effective and safe in both children and adults^43^,^44^,^45^,^46^,^47^. Its active principle, an extract of bacterial lysates isolated from 21 known respiratory pathogenic strains, showed protection against airway infections of bacterial and viral origin. In particular, several studies highlighted the capacity of OM-85 to trigger immunomodulatory and protective immune responses against diverse pathogens *in vivo*^48^,^49^,^50^,^51^,^52^,^53^,^54^, including influenza and respiratory syncytial virus as well bacterial superinfection following influenza^55^,^56^.

Despite these findings, the effects of OM-85 on the production of type I interferons or inflammasome-mediated IL-1 production have not been assessed. We thus dissected the role of OM-85 in regulating these pathways *in vitro*. We found that OM-85 induced IFN-β production and acted as an effective priming agent inducing substantial levels of proIL-1α and β. Surprisingly, OM-85 also interfered with inflammasome activity specifically in response to alum. These results suggest that OM-85 dampens selected inflammatory reactions, while potentiating innate immune defenses, two effects that might contribute to reducing infections and alleviating COPD and asthmatic conditions in the context of the respiratory tract.

## Results

### OM-85 induces interferon-β production

**Figure 1:**
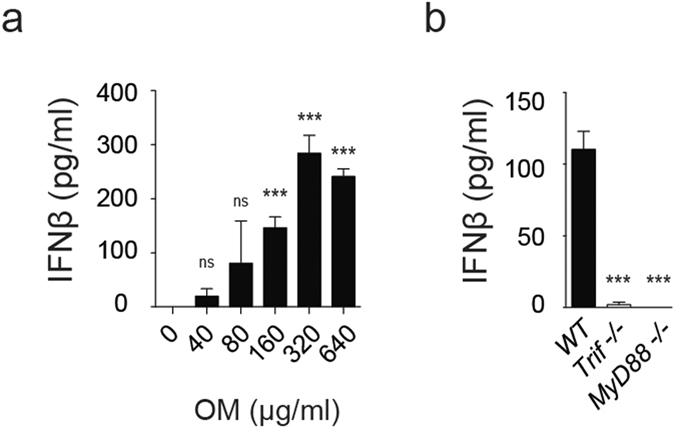
OM-85 promotes the production of type I IFN. (**a** and **b**) Wild type (WT) BMDCs were stimulated with the indicated doses of OM-85 (**a**) or Wild type (WT), *Trif*^−/−^, and *MyD88*^−/−^ BMDCs were stimulated with 320 μg/ml OM-85 (**b**) for 16 hours (h). IFN-β secretion was measured by ELISA. Results represent mean ± SD (n = 3 technical replicates) and are representative of at least four (**a**) and two (**b**) independent experiments. Statistical differences were calculated between the condition without OM-85 (0) and each of the following doses using Student’s t-test adjusted by Bonferroni correction over 5 (**a**) and between the wild type condition and the mutant genotypes using Student’s t-test adjusted by Bonferroni correction over 2 (**b**). ns, non-significant; ***p ≤ 0.001.

Given the potential relevance of type I IFN in asthmatic patients, we investigated the ability of OM-85 to induce this cytokine. We focused on DCs, which are proficient in producing type I IFNs and in activating the inflammasome pathway^21^,^25^,^26^. We therefore treated bone marrow-derived DCs (BMDCs) for 16 hours and assessed IFN-β release in the supernatant. IFN-β production was detected and followed a bell-shaped curve as a function of OM-85 concentration (Fig. 1a). To gain insights into the OM-85-dependent mechanisms inducing IFN-β, we took advantage of *Trif*- and *MyD88*-deficient BMDCs. Interestingly, the absence of either *Trif* or *MyD88* drastically affected OM-85-driven IFN-β production (Fig. 1b). Taken together, we show here that OM-85 has the ability to induce IFN-β production, an important cytokine playing immunomodulatory and antiviral effects, in a MyD88- and Trif-dependent manner.

### OM-85 does per se not lead to IL-1 release

**Figure 2:**
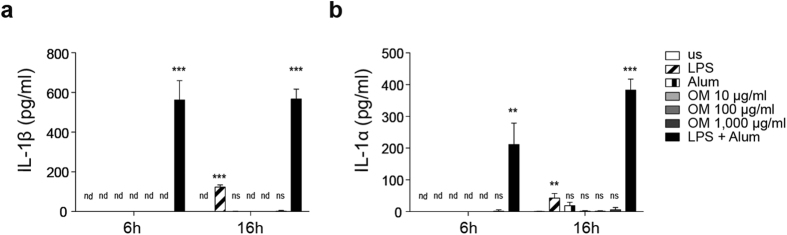
OM-85 does not activate the inflammasome. (**a** and **b**) Dose-response and time course (6 and 16 h) for the release of IL-1β (**a**) and IL-1α (**b**) by BMDCs in response to the indicated concentrations of OM-85 as measured by ELISA. As control, stimulation was performed with LPS (100 ng/ml) for 6 and 16 h or LPS (100 ng/ml) followed by addition of alum (300 μg/ml) for the last 2.5 h. Results represent mean ± SD (n = 3 technical replicates) and are representative of two independent experiments. us = unstimulated (**a** and **b**). Statistical differences were calculated between the unstimulated condition and the other conditions by Student’s t-test adjusted by Bonferroni correction over 6 (**a** and **b**). nd, non-detected; ns, non-significant; **p ≤ 0.01; ***p ≤ 0.001.

We next tested the effects of OM-85 on the inflammasome/IL-1 axis. Given the complex nature of OM-85, we first asked whether this extract exhibited the capacity to induce IL-1β and α production. This encompasses the steps of inducing their precursor forms, referred to as “priming”, and of activating the inflammasome for their cleavage and/or secretion. We therefore treated BMDCs with increasing doses of OM-85 for either 6 or 16 hours and measured the release of IL-1β and IL-1α by ELISA. As shown in Fig. 2a and b, no substantial production of these cytokines was observed as compared to the unstimulated condition, whereas they were nicely released upon standard two-step stimulation performed by LPS-mediated priming and the NLRP3 inflammasome activator aluminium (alum). This indicates that OM-85 has not *per se* the capacity to induce the inflammasome/IL-1 cascade.

### OM-85 acts as a priming signal mainly through MyD88

**Figure 3:**
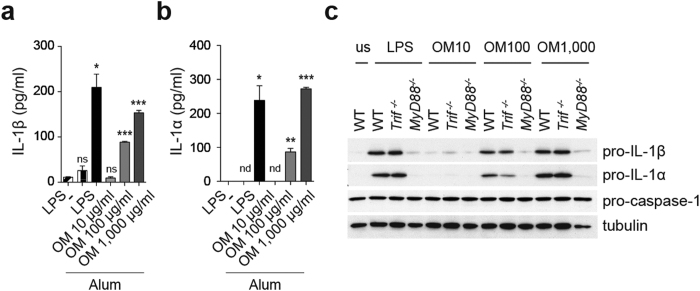
OM-85 is a priming signal for alum-dependent inflammasome activation. (**a** and **b**) BMDCs were primed with 10 ng/ml LPS or the indicated doses of OM-85 for 4 h, followed by inflammasome activation with alum (300 μg/ml) for 2.5 h. Release of IL-1β (**a**) and IL-1α (**b**) was measured by ELISA. Results represent mean ± SD (n = 3 technical replicates) and are representative of at least four independent experiments (**a** and **b**). Statistical differences were calculated between the LPS only condition and the other conditions by Student’s t-test adjusted by Bonferroni correction over 5 (**a** and **b**). nd, non-detected; ns, non-significant; *p ≤ 0.05; **p ≤ 0.01; ***p ≤ 0.001. Wild type (WT), *Trif*^−/−^, and *MyD88*^−/−^ (**c**) BMDCs were primed with 10 ng/ml LPS or the indicated doses of OM-85 for 4 h. Levels of proIL-1β, proIL-1α, pro-caspase 1, and tubulin as control, were assessed in cell extracts by immunoblot analysis. Results are representative of two (**c**) independent experiments. us = unstimulated.

Despite the inability to induce IL-1 secretion, the bacterial origin of OM-85 suggested it to be able to act as a priming signal. We therefore tested its ability to prime BMDCs, which were then treated with alum. As shown in Fig. 3a and b, exposure to OM-85 effectively induced the production of IL-1β and IL-1α by alum-triggered BMDCs. We thus tested the induction of proIL-1β and proIL-1α in BMDCs upon a four hours treatment with OM-85. As shown in Fig. 3c, the bacterial extract increased the amount of proIL-1α and proIL-1β in a dose-dependent manner.

Next, we used *Trif*- and *MyD88*-deficient BMDCs to understand whether these adaptor proteins mediated OM-85-induced proIL-1β and proIL-1α. Whereas the absence of *Trif* only slightly affected the levels of proIL-1α, *MyD88* deficiency abolished OM-85-induced priming with regard to both proIL-1β and proIL-1α (Fig. 3c). These data indicate that OM-85 did not deploy toxic effects even at high concentrations and was active, modulating pro-IL-1β and α in a MyD88-dependent manner.

### OM-85 acts as a priming signal for the NLRP3 and the AIM2 inflammasomes

**Figure 4:**
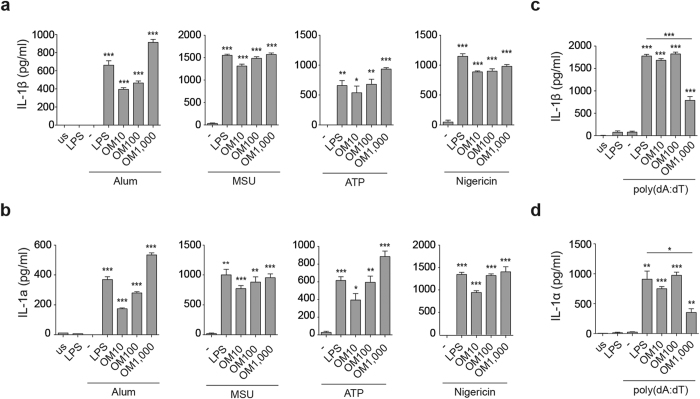
OM-85 is a priming signal for multiple inflammasomes. (**a** and **b**) BMDCs were primed with 10 ng/ml LPS or the indicated doses of OM-85 (referring to μg/ml) for 4 h, followed by stimulation with alum (300 μg/ml), MSU (300 μg/ml), nigericin, (0.1 μM) for 2.5 h and ATP (500 μM) for 45 min. Release of IL-1β and IL-1α was measured by ELISA. (**c** and **d**) BMDCs were primed as described above, followed by transfection of poly(dA:dT) (0.6 µg). 6 h after, release of IL-1β (**c**) and IL-1α (**d**) in the supernatants was measured by ELISA. Results represent mean ± SD (n = 3 technical replicates) and are representative of at least three (**a** and **b**) and two (**c** and **d**) independent experiments. us = unstimulated. Statistical differences were calculated between the condition exposed to the inflammasome activator only (Alum, MSU, ATP, or Nigericin in the absence of LPS) and the conditions treated with LPS or OM-85 and the inflammasome activator by Student’s t-test adjusted by Bonferroni correction over 4 (**a** and **b**). Statistical differences were calculated between the condition exposed to poly(dA:dT) in the absence of LPS and the conditions treated with LPS or OM-85 and poly(dA:dT) as well as between the condition treated by LPS and poly(dA:dT) and by OM-85 (1000 μg/ml) and poly(dA:dT) using Student’s t-test adjusted by Bonferroni correction over 5 (**c** and **d**). *p ≤ 0.05; **p ≤ 0.01; ***p ≤ 0.001.

We next asked whether OM-85 was an effective priming agent for multiple activators of the NLRP3 as well as for the AIM2 inflammasome, as both these platforms are important for detection of and protection from viral infections. To address these questions, BMDCs pre-treated for four hours with different doses of OM-85 were exposed to the particulate NLRP3 activators alum and monosodium urate (MSU) crystals as well as to the soluble activators ATP and nigericin. As illustrated in Fig. 4a and b, OM-85 was an efficient priming signal for all of these stimuli. While the effects by OM-85 treatment were consistent in all experiments, it is important to mention that the activity of OM-85 on BMDC was subject to some variation, as illustrated here between panels 3a/b and 4a/b at the lower doses applied (e.g. 10 ug/ml).

In addition, we observed that OM-85 acted in the same way for the AIM2 inflammasome (Fig. 4c and d). Intriguingly however, BMDCs pre-treated with the highest tested dose of OM-85 showed reduced production of IL-1α and IL-1β upon polydA:dT-mediated activation.

### OM-85 pre-treatment interferes with LPS and alum-induced inflammasome activation

**Figure 5:**
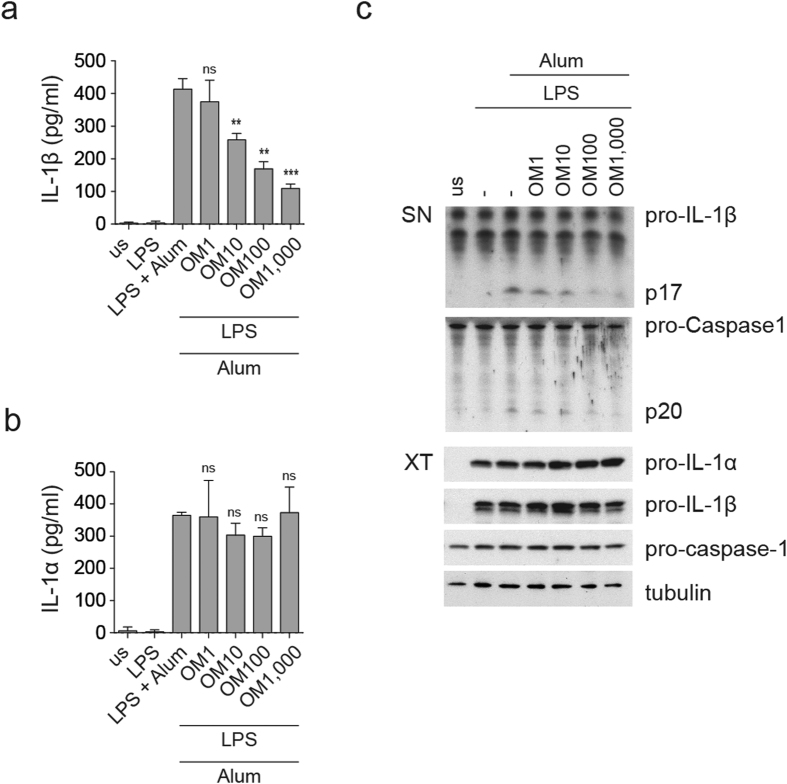
OM-85 dampens IL-1β release induced by LPS priming and alum. (**a**–**c**) BMDCs were pre-treated overnight with the indicated doses of OM-85 (μg/ml), primed with 10 ng/ml LPS for 4 h and then treated with 300 μg/ml alum for 2.5 h. Release of IL-1β (**a**) and IL-1α (**b**) was measured by ELISA. Results represent mean ± SD (n = 3 technical replicates) (**a** and **b**). An illustration of all experiments performed is provided in Supplementary Figure 1a. (**c**) Levels of cleaved IL-1β and caspase 1 were assessed in culture supernatant by immunoblot analysis, whereas pro-IL-1β, pro-IL-1α, pro-caspase 1, and tubulin as control, were assessed in cell extracts. Results are representative of at least three independent experiments. Statistical significance was calculated between the condition treated with LPS and alum and the conditions pre-treated with various doses of OM-85 using Student’s t-test adjusted by Bonferroni correction over 4 (**a**,**b**). ns, non-significant; **p ≤ 0.01; ***p ≤ 0.001.

The observation that high-dose OM-85 induced a decrease of AIM2-mediated IL-1α and IL-1β release prompted us to further explore potential dampening effects of OM-85 on inflammasome activity. We therefore performed a modified version of the inflammasome activation assay in which we pre-treated BMDCs overnight with OM-85 and added LPS for four hours, followed by inflammasome activators. Whereas we did not observe significant inhibitory effects on IL-1α and IL-1β secretion upon inflammasome triggering by ATP, we found that IL-1β secretion induced by alum and – to a smaller extent by MSU – was downmodulated by OM-85 pre-treatment (Fig. 5a and Supplementary Figure 1a). However, secreted IL-1α was not substantially decreased, as shown in Fig. 5b. In order to explain the reduced release of IL-1β, we checked the levels of cleaved caspase-1. Interestingly, caspase-1 activation was decreased by overnight OM-85 pre-treatment (Fig. 5c and Supplementary Figure 1b). Taken together, these data suggests that OM-85 pre-treatment dampens IL-1β production, at least partly through reducing inflammasome activity in response to alum.

### Alum-driven peritonitis is reduced by OM-85

**Figure 6:**
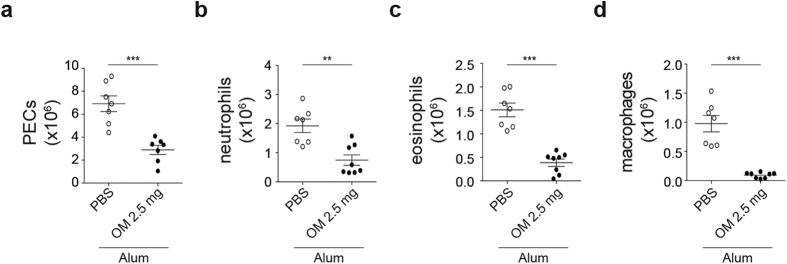
OM-85 hinders recruitment of inflammatory cells upon alum-induced peritoneal inflammation. (**a**–**d**) C57BL/6 mice were injected intravenously with the indicated dose of OM-85 or PBS as a control. 5 h later, mice were challenged intraperitoneally with 350 μg of alum. 12 h after alum injection, mice were sacrificed and peritoneal lavages were performed. Absolute numbers of peritoneal exudate cells (PECs) (**a**), neutrophils (**b**), eosinophils (**c**), and macrophages (**d**) recruited to the peritoneal cavity are depicted. Results represent mean ± SEM (n = 7 mice per group) and are a pool of two experiments and are representative of four independent experiments. Statistical significance between control and OM-85-treated mice was calculated using Student’s t-test. **p ≤ 0.01; ***p ≤ 0.001.

Based on the previous finding, we then tested whether the immunomodulatory effects of OM-85 on the inflammasome were recapitulated in an *in vivo* model. We focused on the well-established alum-dependent peritonitis model, which is mediated by IL-1α and IL-1β and is commonly used to assess inflammasome activity *in vivo*^19^. Wild type mice were pre-treated intravenously with OM-85, or PBS as control and, five hours later, injected intraperitoneally (i.p.) with alum. Twelve hours post alum-injection, mice were sacrificed and peritoneal exudate cells analyzed by flow cytometry. As depicted in Fig. 6a, a remarkable decrease in the numbers of recruited cells was observed. In addition, when specifically assessing the abundance of individual inflammatory subsets, a significant decrease in the numbers of neutrophils, eosinophils, as well as macrophages was observed (Fig. 6b–d). These data indicate that OM-85 has the ability of interfering also *in vivo* with alum-induced inflammatory reactions.

## Discussion

Here we have provided evidence for the immunomodulatory effects of OM-85 on the production of two cytokines playing key roles in lung infections and diseases: type I interferons and IL-1. We found that OM-85 was able to induce IFN-β at an optimal intermediate concentration but less at higher doses. Interestingly, these data are reminiscent of results obtained with other inducers of type I IFNs, including DNA, RNA, and fungal pathogen-derived molecular patterns, which show a bell-shaped dose-response curve^57^,^58^,^59^,^60^. These findings suggested that OM-85-triggered IFN production was compatible with TLR stimulation, a hypothesis that was corroborated by the dependency on both TLR adaptor proteins Trif and MyD88. The ability of OM-85 to induce type I IFN was also observed – to a smaller extent – in human DCs^61^. This represents an important feature potentially involved in the beneficial effects of OM-85 in airway inflammatory diseases, as IFN is a well-known antiviral cytokine, contributing to minimize the occurrence of various respiratory tract infections and the subsequent exacerbations of chronic inflammatory conditions^62^,^63^.

Whereas OM-85 did not activate the inflammasome *per se*, it nicely primed BMDCs, significantly rising proIL-1α and proIL-1β levels, in agreement with data in human DCs^61^. Furthermore, our results showed that MyD88 was necessary to augment the levels of proIL-1α and proIL-1β, which are canonical NF-κB targets, downstream of OM-85. Along these lines, previous findings showed a central role for TLRs, MyD88, and activation of downstream NF-κB signaling by OM-85^48^,^51^,^61^. In addition, we show here the involvement of the adaptors MyD88 and Trif in the induction of type I IFN, inferring the implication of TLR3 or TLR4 upon OM-85 treatment^51^.

In this work we focused on two inflammasome platforms relevant in the context of antiviral responses, and, in particular, of airway infections: AIM2 and NLRP3^16^,^23^,^24^,^25^,^26^,^27^. We showed that OM-85 exposure efficiently pre-activated BMDCs, an effect that was also demonstrated for *proIL-1β* transcript level on human DCs, for subsequent inflammasome activation and IL-1 production^61^. Given the role of the NLRP3 inflammasome and recent data proving the efficacy of OM-85 in controlling influenza infection, it is conceivable that part of the protective effect of OM-85 is mediated by priming of the inflammasome–IL-1 axis^16^,^23^,^24^,^25^,^26^,^27^,^56^. Our results indicate therefore that OM-85 treatment keeps innate immune cells in an “alerted state” ideal to release large amounts of IL-1 upon sensing an inflammasome trigger and, conceivably, to reduce selected viral infections.

Yet, the pre-activated state induced by OM-85 in BMDCs is not “inflammatory” *per se*, as negligible release of IL-1 is measured under these conditions. This is an important aspect when considering the detrimental effects of IL-1 on chronic inflammatory diseases^1^. It therefore seems that OM-85 has the ability to boost inflammation, but exclusively when specific activators of the inflammasome are present. Interestingly, we noticed that the effects of OM-85 on priming, particularly at low doses, diverged in response to different inflammasome activators. In general, activators inducing lower levels of IL-1 release, such as alum, required higher doses of OM-85, indicating a stronger dependency on priming.

Interestingly, we also observed that an overnight pre-treatment with OM-85 dampened the release of IL-1β induced by canonical LPS and alum stimulation. This inhibitory activity was in part due to a reduced activation of caspase-1. These *in vitro* results were corroborated by *in vivo* data showing that OM-85 pre-treatment substantially interferes with an IL-1-driven peritonitis model. It is important to stress that *in vivo* i.p. treatment with alum is sufficient for the secretion of IL-1α and IL-1β, indicating that their precursor proteins are synthesized in response to endogenous priming stimuli. As we showed, OM-85 was *per se* able to pre-activate BMDCs for inflammasome activation and IL-1 production, suggesting therefore that its immunomodulatory nature renders it activatory in the absence of additional priming signals and – to some extent – inhibitory in their presence. It was however surprising to see that this effect was quite specific to alum and observed upon activation by MSU only when OM-85 was used at very high doses. This indicates that, besides clear differences between soluble and particulate activators^32^, more subtle distinctions exists among the latters, which might reside in their chemical or physical properties, including their size.

Despite a significant body of literature demonstrating that the inflammasome pathway exerts multiple and important functions in the pathophysiology of airway diseases, whether OM-85 regulates such cellular responses remained unanswered. Derived from common human bacterial respiratory tract pathogens and used for the prevention of airway infections and chronic inflammation, OM-85 exhibits the capacity to modulate inflammasome responses. Our data suggest that the mode of action of OM-85 with regard to the IL-1 pathway is twofold. On the one hand, it dampens IL-1β production in response to alum. This effect might be important to prevent selected inflammatory reactions also in the lung, where OM-85 was previously shown to reduce inflammation^48^. On the other hand, OM-85 clearly supports adequate IL-1 production in the case of inflammasome activation. This effect might contribute to the protective effects of OM-85 in the presence of viruses controlled by the inflammasome^56^.

## Experimental procedures

### Immunoblot analysis

The antibody against mouse IL-1β was a gift from R. Solari (Imperial College, London), whereas the antibody against mouse caspase-1 was a gift from P. Vandenabeele (Ghent University, Belgium)^64^,^65^. Other antibodies used were monoclonal anti-tubulin from Sigma-Aldrich; rabbit polyclonal antibody to β-actin from Abcam; anti-IL-1α (ALF161) from ebioscience.

### Mice

Six- to 12-wk-old C57BL/6, *Trif*^−/−^66, and *MyD88*^−/−^67 mice were housed at the animal facility of the University of Lausanne. All animal experimental protocols were approved by the Veterinary office regulations of the State of Vaud, Switzerland, and all methods were performed in accordance with the Swiss guidelines and regulations.

### OM-85 formulation

OM-85 is produced at OM Pharma SA, Meyrin 1/Geneva, Switzerland. Bacteria are grown in individual batches, heat inactivated once they reach a critical mass, harvested, and then subject to alkaline lysis. After lysis, the lysates from the 21 different bacterial strains are mixed together, filtered, and the soluble fraction is neutralized. OM-85 active principle is an alkaline aqueous soluble extract obtained by microfiltration of the pooled 21 bacterial lysates of Haemophilus influenzae (1 strain), Streptococcus pneumoniae (4 strains), Klebsiella pneumoniae subsp. pneumoniae (2 strains), Klebsiella pneumoniae subsp. ozaenae (1 strains), Staphylococcus aureus (6 strains), Streptococcus pyogenes (1 strains), Streptococcus sanguinis (3 strains), Moraxella (Branhamella) catarrhalis (3 strains) with the following characteristics: 24 mg dry weight bacterial extract per mL. The oral formulation is the isotonic OM-85 bacterial extract neutralized at pH 7.0 using HCl 1 M.

### *In vitro* stimulation experiments

BMDCs were differentiated as previously described^68^. For normal stimulation experiments, 7 × 10^4^ differentiated BMDCs were plated in DC differentiation medium (RPMI 1640, 10% FCS, 100 U/ml penicillin, 100 μg/ml streptomycin, 50 μN 2-ME, 10 mM HEPES, supplemented with 20 ng/ml rGM-CSF (ImmunoTools)) and treated for the indicated times and with the indicated doses of OM-85 of ultrapure LPS (Invivogen). For inflammasome activation experiments, 7 × 10^4^ differentiated BMDCs were primed with 10 ng/ml ultrapure LPS or the indicated doses of OM-85 for the 4 h preceding inflammasome stimulations or left unprimed. Then, stimulations were carried out. ATP (500 μM) and nigericin (0.1 μM) were from Sigma, MSU crystals (300 μg/ml) were from Adipogen, and alum (300 μg/ml) from Pierce Biochemicals (Imject-alum). ATP stimulations were performed for 45 min and other stimulations for 150 min. For the stimulation of the AIM2 inflammasome, poly(dA:dT) (purchased from Invivogen) was admixed at the indicated concentrations to Lipofectamine 2000 (from Invitrogen) according to manufacturer’s instructions and cells were stimulated for 6 h. For inflammasome inhibition experiments, 7 × 10^4^ differentiated BMDCs were incubated for 12 h or the indicated times in the presence of OM-85. Cells were then primed with 10 ng/ml ultrapure LPS for the 4 h preceding inflammasome stimulations. Then, stimulations were carried out with the doses of inflammasome activators indicated in the text.

### *In vivo* peritonitis experiments

For peritonitis, mice were injected i.v., with 2.5 mg OM85 followed 5 h later by an i.p. injection of 350 μg alum (Pierce). 12–14 h after alum injection, mice were sacrificed and peritoneal cavities were washed with 6 ml PBS. PECs were counted and analyzed by flow cytometry using a combination of antibodies against CD11b (M1/70), Ly6C (AL-21), Ly6G (1A8) (BD Pharmingen), antiCD16/32 (93), CD11c (N418), F4/80 (BM8) (eBioscience). For cellular subtype analysis the following gating strategies were used: neutrophils (CD11b^+^, Ly6G^high^, F4/80^−^), eosinophils (CD11b^+^, SSC^high^) and macrophages (Ly6G^−^, SSC^low^, CD11b^+^, F4/80^+^), recruitment was analyzed on a FACSCanto (BD Bioscience) by using the FLOWJO software (Tree Star).

### ELISA

Cell culture supernatants were assayed for mouse IL-1β, IL-1α (ebioscience), and IFN-β (PBL Assay Science) according to manufacturer’s instructions.

### Statistical analysis

Statistical analyses were calculated as described in the Figure legends (GraphPad Prism version 5.0).

## Additional Information

**How to cite this article:** Dang, A. T. *et al*. OM-85 is an immunomodulator of interferon-β production and inflammasome activity. *Sci. Rep.* **7**, 43844; doi: 10.1038/srep43844 (2017).

**Publisher's note:** Springer Nature remains neutral with regard to jurisdictional claims in published maps and institutional affiliations.

## Supplementary Material

Supplementary Information

## References

[b1] Barnes P. J. The cytokine network in asthma and chronic obstructive pulmonary disease. The Journal of clinical investigation 118, 3546–3556 (2008). 18982161 10.1172/JCI36130 PMC2575722

[b2] Wark P. A. . Asthmatic bronchial epithelial cells have a deficient innate immune response to infection with rhinovirus. The Journal of experimental medicine 201, 937–947 (2005). 15781584 10.1084/jem.20041901 PMC2213100

[b3] Contoli M. . Role of deficient type III interferon-lambda production in asthma exacerbations. Nature medicine 12, 1023–1026 (2006). 10.1038/nm1462 16906156

[b4] Platanias L. C. Mechanisms of type-I- and type-II-interferon-mediated signalling. Nature reviews. Immunology 5, 375–386 (2005). 10.1038/nri1604 15864272

[b5] Ludigs K., Parfenov V., Du Pasquier R. A. & Guarda G. Type I IFN-mediated regulation of IL-1 production in inflammatory disorders. Cell Mol Life Sci 69, 3395–3418 (2012). 22527721 10.1007/s00018-012-0989-2 PMC11115130

[b6] Simon H. U., Seelbach H., Ehmann R. & Schmitz M. Clinical and immunological effects of low-dose IFN-alpha treatment in patients with corticosteroid-resistant asthma. Allergy 58, 1250–1255 (2003). 14616099 10.1046/j.1398-9995.2003.00424.x

[b7] Kumagai Y. . Alveolar macrophages are the primary interferon-alpha producer in pulmonary infection with RNA viruses. Immunity 27, 240–252 (2007). 17723216 10.1016/j.immuni.2007.07.013

[b8] Shahangian A. . Type I IFNs mediate development of postinfluenza bacterial pneumonia in mice. The Journal of clinical investigation 119, 1910–1920 (2009). 19487810 10.1172/JCI35412 PMC2701856

[b9] Goritzka M. . Alveolar macrophage-derived type I interferons orchestrate innate immunity to RSV through recruitment of antiviral monocytes. The Journal of experimental medicine 212, 699–714 (2015). 25897172 10.1084/jem.20140825 PMC4419339

[b10] Meissner N., Swain S., McInnerney K., Han S. & Harmsen A. G. Type-I IFN signaling suppresses an excessive IFN-gamma response and thus prevents lung damage and chronic inflammation during Pneumocystis (PC) clearance in CD4 T cell-competent mice. The American journal of pathology 176, 2806–2818 (2010). 20395428 10.2353/ajpath.2010.091158 PMC2877842

[b11] Arimori Y. . Type I interferon limits influenza virus-induced acute lung injury by regulation of excessive inflammation in mice. Antiviral research 99, 230–237 (2013). 23721943 10.1016/j.antiviral.2013.05.007

[b12] Zdrenghea M. T., Makrinioti H., Muresan A., Johnston S. L. & Stanciu L. A. The role of macrophage IL-10/innate IFN interplay during virus-induced asthma. Reviews in medical virology 25, 33–49 (2015). 25430775 10.1002/rmv.1817 PMC4316183

[b13] Martinez F. D. The connection between early life wheezing and subsequent asthma: The viral march. Allergologia et immunopathologia 37, 249–251 (2009). 19875225 10.1016/j.aller.2009.06.008

[b14] Sly P. D., Kusel M. & Holt P. G. Do early-life viral infections cause asthma? The Journal of allergy and clinical immunology 125, 1202–1205 (2010). 20304476 10.1016/j.jaci.2010.01.024

[b15] Murray C. S., Simpson A. & Custovic A. Allergens, viruses, and asthma exacerbations. Proceedings of the American Thoracic Society 1, 99–104 (2004). 16113420 10.1513/pats.2306027

[b16] De Nardo D., De Nardo C. M. & Latz E. New insights into mechanisms controlling the NLRP3 inflammasome and its role in lung disease. The American journal of pathology 184, 42–54 (2014). 24183846 10.1016/j.ajpath.2013.09.007 PMC3873477

[b17] Ather J. L., Martin R. A., Ckless K. & Poynter M. E. Inflammasome Activity in Non-Microbial Lung Inflammation. Journal of environmental immunology and toxicology 1, 108–117 (2014). 25642415 PMC4308734

[b18] Schroder K. & Tschopp J. The inflammasomes. Cell 140, 821–832 (2010). 20303873 10.1016/j.cell.2010.01.040

[b19] Gross O., Thomas C. J., Guarda G. & Tschopp J. The inflammasome: an integrated view. Immunological reviews 243, 136–151 (2011). 21884173 10.1111/j.1600-065X.2011.01046.x

[b20] Rathinam V. A. & Fitzgerald K. A. Inflammasome Complexes: Emerging Mechanisms and Effector Functions. Cell 165, 792–800 (2016). 27153493 10.1016/j.cell.2016.03.046 PMC5503689

[b21] Guarda G. . Differential Expression of NLRP3 among Hematopoietic Cells. Journal of immunology 186, 2529–2534 (2011). 10.4049/jimmunol.1002720 21257968

[b22] Xiao T. S. The nucleic acid-sensing inflammasomes. Immunological reviews 265, 103–111 (2015). 25879287 10.1111/imr.12281 PMC4545668

[b23] Lupfer C., Malik A. & Kanneganti T. D. Inflammasome control of viral infection. Current opinion in virology 12, 38–46 (2015). 25771504 10.1016/j.coviro.2015.02.007 PMC4470791

[b24] Allen I. C. . The NLRP3 inflammasome mediates *in vivo* innate immunity to influenza A virus through recognition of viral RNA. Immunity 30, 556–565 (2009). 19362020 10.1016/j.immuni.2009.02.005 PMC2803103

[b25] Thomas P. G. . The intracellular sensor NLRP3 mediates key innate and healing responses to influenza A virus via the regulation of caspase-1. Immunity 30, 566–575 (2009). 19362023 10.1016/j.immuni.2009.02.006 PMC2765464

[b26] Ichinohe T., Lee H. K., Ogura Y., Flavell R. & Iwasaki A. Inflammasome recognition of influenza virus is essential for adaptive immune responses. The Journal of experimental medicine 206, 79–87 (2009). 19139171 10.1084/jem.20081667 PMC2626661

[b27] McAuley J. L. . Activation of the NLRP3 inflammasome by IAV virulence protein PB1-F2 contributes to severe pathophysiology and disease. PLoS pathogens 9, e1003392 (2013). 23737748 10.1371/journal.ppat.1003392 PMC3667782

[b28] Gasse P. . IL-1R1/MyD88 signaling and the inflammasome are essential in pulmonary inflammation and fibrosis in mice. The Journal of clinical investigation 117, 3786–3799 (2007). 17992263 10.1172/JCI32285 PMC2066195

[b29] Gasse P. . Uric acid is a danger signal activating NALP3 inflammasome in lung injury inflammation and fibrosis. American journal of respiratory and critical care medicine 179, 903–913 (2009). 19218193 10.1164/rccm.200808-1274OC

[b30] Kuipers M. T. . Ventilator-induced lung injury is mediated by the NLRP3 inflammasome. Anesthesiology 116, 1104–1115 (2012). 22531249 10.1097/ALN.0b013e3182518bc0

[b31] Dostert C. . Innate immune activation through Nalp3 inflammasome sensing of asbestos and silica. Science 320, 674–677 (2008). 18403674 10.1126/science.1156995 PMC2396588

[b32] Hornung V. . Silica crystals and aluminum salts activate the NALP3 inflammasome through phagosomal destabilization. Nature immunology 9, 847–856 (2008). 18604214 10.1038/ni.1631 PMC2834784

[b33] Dostert C., Ludigs K. & Guarda G. Innate and adaptive effects of inflammasomes on T cell responses. Current opinion in immunology 25, 359–365 (2013). 23478069 10.1016/j.coi.2013.02.008

[b34] Okada S. . Potential role of interleukin-1 in allergen-induced late asthmatic reactions in guinea pigs: suppressive effect of interleukin-1 receptor antagonist on late asthmatic reaction. J Allergy Clin Immunol 95, 1236–1245 (1995). 7797792 10.1016/s0091-6749(95)70081-1

[b35] Schmitz N., Kurrer M. & Kopf M. The IL-1 receptor 1 is critical for Th2 cell type airway immune responses in a mild but not in a more severe asthma model. Eur J Immunol 33, 991–1000 (2003). 12672065 10.1002/eji.200323801

[b36] Nakae S. . IL-1 is required for allergen-specific Th2 cell activation and the development of airway hypersensitivity response. Int Immunol 15, 483–490 (2003). 12663678 10.1093/intimm/dxg054

[b37] Li T. . Pharmacokinetics and anti-asthmatic potential of non-parenterally administered recombinant human interleukin-1 receptor antagonist in animal models. J Pharmacol Sci 102, 321–330 (2006). 17116976 10.1254/jphs.fpj06007x

[b38] Kool M., Fierens K. & Lambrecht B. N. Alum adjuvant: some of the tricks of the oldest adjuvant. J Med Microbiol 61, 927–934 (2012). 22174375 10.1099/jmm.0.038943-0

[b39] Allen I. C. . Analysis of NLRP3 in the development of allergic airway disease in mice. Journal of immunology 188, 2884–2893 (2012). 10.4049/jimmunol.1102488 PMC3294123 22323538

[b40] Holt P. G. . Drug development strategies for asthma: in search of a new paradigm. Nature immunology 5, 695–698 (2004). 15224096 10.1038/ni0704-695

[b41] Spellberg B., Bartlett J. G. & Gilbert D. N. The future of antibiotics and resistance. The New England journal of medicine 368, 299–302 (2013). 23343059 10.1056/NEJMp1215093 PMC3617123

[b42] Karchmer A. W. Increased antibiotic resistance in respiratory tract pathogens: PROTEKT US–an update. Clinical infectious diseases: an official publication of the Infectious Diseases Society of America 39 Suppl 3, S142–150 (2004). 15546109 10.1086/421352

[b43] Paupe J. Immunotherapy with an oral bacterial extract (OM-85 BV) for upper respiratory infections. Respiration; international review of thoracic diseases 58, 150–154 (1991). 1745846 10.1159/000195916

[b44] Collet J. P. . Stimulation of nonspecific immunity to reduce the risk of recurrent infections in children attending day-care centers. The Epicreche Research Group. The Pediatric infectious disease journal 12, 648–652 (1993). 8414777 10.1097/00006454-199308000-00005

[b45] Schaad U. B., Mutterlein R., Goffin H. & Group B. V.-C. S. Immunostimulation with OM-85 in children with recurrent infections of the upper respiratory tract: a double-blind, placebo-controlled multicenter study. Chest 122, 2042–2049 (2002). 12475845 10.1378/chest.122.6.2042

[b46] Razi C. H. . The immunostimulant OM-85 BV prevents wheezing attacks in preschool children. The Journal of allergy and clinical immunology 126, 763–769 (2010). 20920766 10.1016/j.jaci.2010.07.038

[b47] Schaad U. B. OM-85 BV, an immunostimulant in pediatric recurrent respiratory tract infections: a systematic review. World journal of pediatrics: WJP 6, 5–12 (2010). 20143206 10.1007/s12519-010-0001-x

[b48] Navarro S. . The oral administration of bacterial extracts prevents asthma via the recruitment of regulatory T cells to the airways. Mucosal immunology 4, 53–65 (2011). 20811345 10.1038/mi.2010.51

[b49] Fu R. . Broncho-Vaxom attenuates allergic airway inflammation by restoring GSK3beta-related T regulatory cell insufficiency. PloS one 9, e92912 (2014). 24667347 10.1371/journal.pone.0092912 PMC3965496

[b50] Han L. . A bacterial extract of OM-85 Broncho-Vaxom prevents allergic rhinitis in mice. American journal of rhinology & allergy 28, 110–116 (2014). 24717947 10.2500/ajra.2013.27.4021

[b51] Luan H. . OM85-BV induced the productions of IL-1beta, IL-6, and TNF-alpha via TLR4- and TLR2-mediated ERK1/2/NF-kappaB pathway in RAW264.7 cells. Journal of interferon & cytokine research: the official journal of the International Society for Interferon and Cytokine Research 34, 526–536 (2014). 10.1089/jir.2013.0077 PMC4080859 24605772

[b52] Roth M. & Block L. H. Distinct effects of Broncho-Vaxom (OM-85 BV) on gp130 binding cytokines. Thorax 55, 678–684 (2000). 10899245 10.1136/thorax.55.8.678 PMC1745835

[b53] Huber M., Mossmann H. & Bessler W. G. Th1-orientated immunological properties of the bacterial extract OM-85-BV. European journal of medical research 10, 209–217 (2005). 15946922

[b54] Scott N. M. . Protection against maternal infection-associated fetal growth restriction: proof-of-concept with a microbial-derived immunomodulator. Mucosal immunology(2016). 10.1038/mi.2016.85 27759021

[b55] Bessler W. G., Vor dem Esche U. & Masihi N. The bacterial extract OM-85 BV protects mice against influenza and Salmonella infection. International immunopharmacology 10, 1086–1090 (2010). 20601184 10.1016/j.intimp.2010.06.009

[b56] Pasquali C. . Enhanced Mucosal Antibody Production and Protection against Respiratory Infections Following an Orally Administered Bacterial Extract. Frontiers in medicine 1, 41 (2014). 25593914 10.3389/fmed.2014.00041 PMC4292070

[b57] Edwards S. . TLR7 stimulation of APCs results in inhibition of IL-5 through type I IFN and Notch signaling pathways in human peripheral blood mononuclear cells. Journal of immunology 190, 2585–2592 (2013). 10.4049/jimmunol.1200780 23382558

[b58] Biondo C. . Recognition of yeast nucleic acids triggers a host-protective type I interferon response. European journal of immunology 41, 1969–1979 (2011). 21480215 10.1002/eji.201141490

[b59] Hemmi H., Kaisho T., Takeda K. & Akira S. The roles of Toll-like receptor 9, MyD88, and DNA-dependent protein kinase catalytic subunit in the effects of two distinct CpG DNAs on dendritic cell subsets. Journal of immunology 170, 3059–3064 (2003). 10.4049/jimmunol.170.6.3059 12626561

[b60] Waibler Z. . Excessive CpG 1668 stimulation triggers IL-10 production by cDC that inhibits IFN-alpha responses by pDC. European journal of immunology 38, 3127–3137 (2008). 18991289 10.1002/eji.200838184

[b61] Parola C. . Selective activation of human dendritic cells by OM-85 through a NF-kB and MAPK dependent pathway. PloS one 8, e82867 (2013). 24386121 10.1371/journal.pone.0082867 PMC3875422

[b62] Kim E. Y. . Persistent activation of an innate immune response translates respiratory viral infection into chronic lung disease. Nature medicine 14, 633–640 (2008). 10.1038/nm1770 PMC2575848 18488036

[b63] Ahanchian H., Jones C. M., Chen Y. S. & Sly P. D. Respiratory viral infections in children with asthma: do they matter and can we prevent them? BMC pediatrics 12, 147 (2012). 22974166 10.1186/1471-2431-12-147 PMC3471019

[b64] Van de Craen M., Declercq W., Van den brande I., Fiers W. & Vandenabeele P. The proteolytic procaspase activation network: an *in vitro* analysis. Cell death and differentiation 6, 1117–1124 (1999). 10578181 10.1038/sj.cdd.4400589

[b65] Guarda G. . Type I interferon inhibits interleukin-1 production and inflammasome activation. Immunity 34, 213–223 (2011). 21349431 10.1016/j.immuni.2011.02.006

[b66] Yamamoto M. . Role of adaptor TRIF in the MyD88-independent toll-like receptor signaling pathway. Science 301, 640–643 (2003). 12855817 10.1126/science.1087262

[b67] Adachi O. . Targeted disruption of the MyD88 gene results in loss of IL-1- and IL-18-mediated function. Immunity 9, 143–150 (1998). 9697844 10.1016/s1074-7613(00)80596-8

[b68] Guarda G. . T cells dampen innate immune responses through inhibition of NLRP1 and NLRP3 inflammasomes. Nature 460, 269–273 (2009). 19494813 10.1038/nature08100

