# Supplementary material for: OM-85 is an immunomodulator of interferon-β production and inflammasome activity
Source: Sci Rep. 2017 Mar 6;7:43844. doi: 10.1038/srep43844 (PMC5338315; doi:10.1038/srep43844)
Supplement: Supplementary Information [file srep43844-s1.pdf]

Supplementary information for:

OM-85 is an immunomodulator of interferon-β production and inflammasome activity

A.T. Dang[1], C. Pasquali[2], K. Ludigs[1,3], and G. Guarda[1,4]

[1]Department of Biochemistry, University of Lausanne, Epalinges, Switzerland

[2]Vifor-Pharma c/o OM Pharma SA, 1217 Meyrin 1/Geneva, Switzerland

[3]current address: Roche Pharma (Schweiz) AG, Schönmattstrasse 2, 4153 Reinach

[4]corresponding author

Supplementary Figure 1

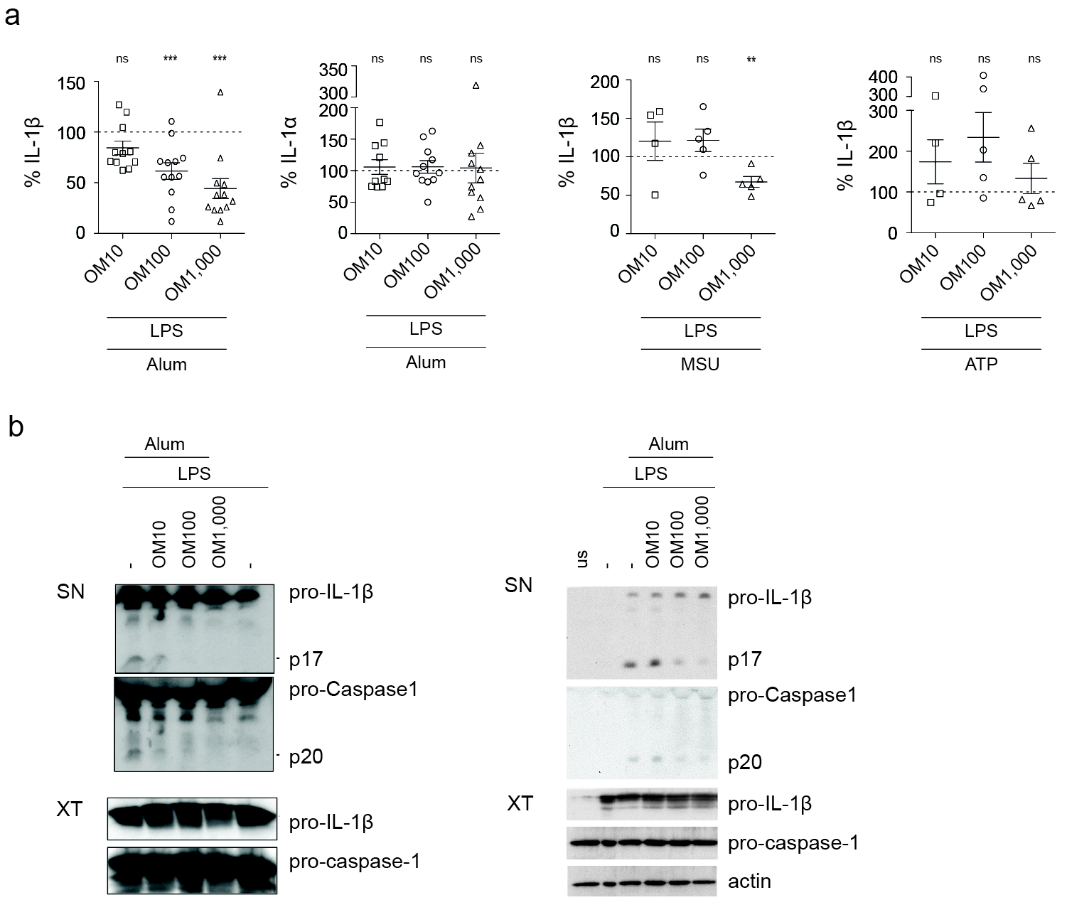

Supplementary Figure 1. OM-85 does not significantly affect IL-1β release induced by LPS priming and MSU or ATP.

(a) BMDCs were pre-treated overnight with the indicated doses of OM-85 (µg/ml), primed with 10 ng/ml LPS for 4 h and then treated with alum (300 µg/ml), MSU (300 µg/ml for 2.5 h, or ATP (500 µM) for 45 min. Release of IL-1β and IL-1α (as indicated) was measured by ELISA. A pool of all performed independent experiments is shown. Released IL-1β (or IL-1α) in the control condition LPS/activator is set as 100 percent (dotted line). The level of secreted IL-1β (or IL-1α) for each OM-85 pre-treated condition is expressed relative to the control. Average and SEM of independent experiments is shown. Statistical significance was calculated between the condition treated with LPS and alum, which was set to 100%, and the conditions pre-treated with various doses of OM-85 using Student's t-test adjusted by Bonferroni correction over 3 (a, b). ns, non-significant; $^{**}p \leq 0.01$; $^{***}p \leq 0.001$. (b) Cleaved IL-1β and caspase 1 were assessed in culture supernatant by immunoblot analysis, whereas pro-IL-1β, pro-caspase 1, and actin as loading control, were assessed in cell extracts. Results are from two independent experiments.
